# Ethyl 2-(2-acetoxy­benzyl­idene)-7-methyl-3-oxo-5-phenyl-2,3-dihydro-5*H*-1,3-thia­zolo[3,2-*a*]pyrimidine-6-carboxyl­ate^1^

**DOI:** 10.1107/S1600536810007853

**Published:** 2010-03-06

**Authors:** Mukesh M. Jotani, Bharat B. Baldaniya, Edward R. T. Tiekink

**Affiliations:** a Department of Physics, Bhavan’s Sheth R.A. College of Science, Ahmedabad, Gujarat 380 001, India; b Department of Chemistry, M.G. Science Institute, Navrangpura, Ahmedabad, Gujarat 380 009, India; c Department of Chemistry, University of Malaya, 50603 Kuala Lumpur, Malaysia

## Abstract

In the title mol­ecule, C_25_H_22_N_2_O_5_S, the atoms of the thia­zolopyrimidine ring system, with the exception of the phenyl-bearing C atom [deviation = 0.177 (2) Å], are essentially planar [r.m.s deviation = 0.100 (2) °] and the mean plane of these atoms forms dihedral angles of 89.86 (10) and 7.97 (8)° with the phenyl and benzene rings, respectively. In the crystal, co-operative C—H⋯O and C—H⋯π inter­actions lead to a supra­molecular chain along the *a* axis. These chains are connected *via* π–π inter­actions [centroid–centroid = 3.7523 (13) Å].

## Related literature

For background to the pharmacological activity of thia­zolo[3,2-a]pyrimidine derivatives, see: Winter *et al.* (1962 ▶); Atwal *et al.* (1990 ▶); Kappe (2000 ▶); Adams *et al.* (2005 ▶). For related structures, see: Jotani & Baldaniya (2007 ▶, 2008 ▶); Baldaniya & Jotani (2008 ▶); Jotani *et al.* (2009 ▶). For additional geometric analysis, see: Cremer & Pople (1975 ▶). Semi-empirical Quantum Chemical Calculations were performed with the *MOPAC2009* program (Stewart, 2009 ▶).
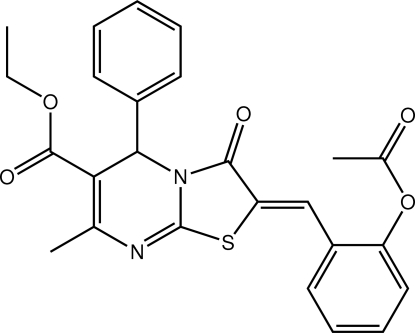


## Experimental

### 

#### Crystal data

C_25_H_22_N_2_O_5_S
*M* *_r_* = 462.51
Triclinic, 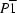

*a* = 8.4236 (3) Å
*b* = 9.6807 (3) Å
*c* = 14.3345 (5) Å
α = 87.939 (2)°
β = 89.680 (2)°
γ = 75.287 (2)°
*V* = 1129.86 (7) Å^3^
*Z* = 2
Mo *K*α radiation
μ = 0.18 mm^−1^
*T* = 293 K
0.47 × 0.35 × 0.20 mm

#### Data collection

Bruker SMART APEX CCD diffractometer
Absorption correction: multi-scan (*SADABS*; Sheldrick, 1996 ▶) *T*_min_ = 0.920, *T*_max_ = 0.965
20701 measured reflections
3969 independent reflections
3438 reflections with *I* > 2σ(*I*)
*R*_int_ = 0.024

#### Refinement

*R*[*F* ^2^ > 2σ(*F* ^2^)] = 0.044
*wR*(*F* ^2^) = 0.137
*S* = 1.06
3969 reflections
301 parameters
H-atom parameters constrained
Δρ_max_ = 0.36 e Å^−3^
Δρ_min_ = −0.31 e Å^−3^

### 

Data collection: *APEX2* (Bruker, 2004 ▶); cell refinement: *APEX2* and *SAINT* (Bruker, 2004 ▶); data reduction: *SAINT* and *XPREP* (Bruker, 2004 ▶); program(s) used to solve structure: *SHELXS97* (Sheldrick, 2008 ▶); program(s) used to refine structure: *SHELXL97* (Sheldrick, 2008 ▶) and *PLATON* (Spek, 2009 ▶); molecular graphics: *ORTEP-3* (Farrugia, 1997 ▶) and *DIAMOND* (Brandenburg, 2006 ▶); software used to prepare material for publication: *publCIF* (Westrip, 2010 ▶).

## Supplementary Material

Crystal structure: contains datablocks global, I. DOI: 10.1107/S1600536810007853/lh5004sup1.cif

Structure factors: contains datablocks I. DOI: 10.1107/S1600536810007853/lh5004Isup2.hkl

Additional supplementary materials:  crystallographic information; 3D view; checkCIF report

## Figures and Tables

**Table 1:** Hydrogen-bond geometry (Å, °) *Cg*1 is the centroid of the S1/C1/N2/C15/C16 ring.

*D*—H⋯*A*	*D*—H	H⋯*A*	*D*⋯*A*	*D*—H⋯*A*
C2—H2⋯O5^i^	0.98	2.58	3.532 (3)	163
C12—H12a⋯*Cg*1^i^	0.97	2.98	3.902 (3)	160

Symmetry code: (i) 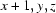.

**Fig. 1.**
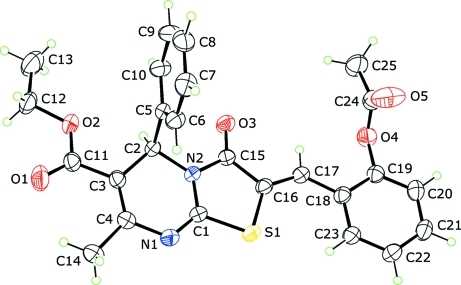
The molecular structure of (I) showing the atom-labelling scheme and displacement ellipsoids at the 35% probability level.

**Fig. 2.**
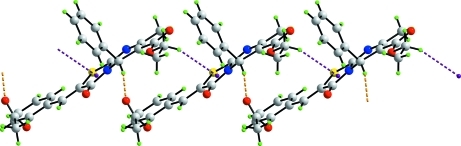
A supramolecular chain aligned along the a axis in (I). The C–H···O and C–H···π contacts are shown as orange and purple dashed lines, respectively. Colour code: S, yellow; O, red; N, blue; C, grey; and H, green.

**Fig. 3.**
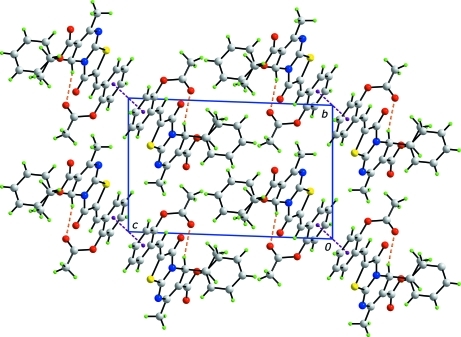
A view in projection down the a axis highlighting the π–π interactions (purple dashed lines) connecting supramolecular chains in (I). The C–H···O contacts are shown as orange dashed lines. Colour code: S, yellow; O, red; N, blue; C, grey; and H, green.

## supplementary crystallographic information

### Comment

The absolute stereochemistry at C2 stereocentre in fused dihydropyrimidine rings is a critical factor for their biological activity and provides an additional opportunity to study the effect of chirality on biological activities (Atwal *et al.*, 1990; Kappe, 2000). The title compound, (I), exhibits anti-cancer (Adams *et al.*, 2005) and anti-inflammatory activities (Winter *et al.*, 1962). In continuation of our structural studies of these pharmacologically interesting thiazolo[3,2-a]pyrimidine derivatives, designed to ascertain the influence of substitution patterns upon crystal packing (Jotani & Baldaniya, 2007; Jotani & Baldaniya, 2008; Baldaniya & Jotani, 2008; Jotani *et al.*, 2009), the synthesis and crystal structure of the title compound, (I), is described herein.

The thiazole ring is essentially planar [maximum deviation of 0.0181 (17) for the N2 atom]. By contrast, the pyrimidine ring is non-planar with the *sp *^3^-C2 atom lying well out of the approximate plane defined by the remaining atoms. The distortion is quantified by the ring puckering parameters (Cremer & Pople, 1975): Q = 0.1721 (19) Å, θ = 67.8 (7) °, and φ_2_ = 164.3 (7) °. The dihedral angle formed between the fused rings is nevertheless small at 6.05 (9) °. The planarity in the molecule extends to the C3-bound acetyl group [the C4–C3–C11–O1 torsion angle is 9.3 (4) °] on one side of the fused-ring system, and on the other through the C16═C17 double bond [1.336 (3) Å] and adjacent benzene ring as seen in the C15–C16–C17–C18 and C16–C17–C18–C19 torsion angles of 174.58 (18) and -174.4 (2) °, respectively. Each of the remaining substituents, *i.e*. the C5–C10 benzene ring and O4-acetyl groups lie to the same side of the molecule and occupy positions defined by the N2–C2–C5–C6 and C18–C19–O4–C24 torsion angles of -64.7 (2) and 94.9 (2) °, respectively. Allowing for the presence of distinct substituents, the molecular framework in (I) resembles closely those found in related derivatives (Jotani & Baldaniya, 2007; Jotani & Baldaniya, 2008; Baldaniya & Jotani, 2008; Jotani *et al.*, 2009)

The crystal structure is stabilised by a variety of weak intermolecular interactions. A supramolecular chain aligned along the *a* axis is formed through the agency of C–H···O and C–H···π interactions, Fig. 2 and Table 1. These are connected via π–π interactions formed between centrosymmetrically related C18–C23 rings [ring centroid(C18–C23)···ring centroid(C18–C23)^i^ = 3.7523 (13) Å for *i*: -*x*, -*y*, -*z*], Fig. 3.

Semi-empirical Quantum Chemical Calculations were performed with the MOPAC2009 program (Stewart, 2009) in order to optimize the experimental structure with the Austin Model 1 (AM1) approximation together with the restricted Hartree-Fock closed-shell wavefunction; minimisations were terminated at a r.m.s. gradient of less than 1.0 kJ mol^-1^ Å^-1^. The heat of formation was calculated to be -313.14 kJ mol^-1^. The ionization potential, dipole moment and self consistency field (SCF) factor were calculated as 8.633 eV, 3.880 Debye, and 121, respectively.

### Experimental

A mixture of ethyl 6-methyl-4-phenyl-2-thioxo-1,2,3,4-tetrahydropyrimidine-5-carboxylate (0.01 mol), chloroacetic acid (0.01 mol), fused sodium acetate (6 g) in glacial acetic acid (25 ml), acetic anhydride (10 ml), and 2-acetyloxy benzaldehyde (0.01 mol) was refluxed for 3 to 3.5 h. The reaction mixture was cooled and poured into cold water. The resulting solid was collected and crystallized from methanol to obtain the final product (75 % yield, m.pt. 438 K). The compound was crystallized by slow evaporation of benzene-ethanol (1:1) solution yielding yellow blocks.

### Refinement

The C-bound H atoms were geometrically placed (C–H = 0.93–0.98 Å) and refined as riding with *U_iso_*(H) = 1.2–1.5*U_eq_*(parent atom).

### Crystal data

**Table:**

C_25_H_22_N_2_O_5_S	*Z* = 2
*M**_r_* = 462.51	*F*(000) = 484
Triclinic, *P*1	*D*_x_ = 1.359 Mg m^−^^3^
Hall symbol: -P 1	Mo *K*α radiation, λ = 0.71073 Å
*a* = 8.4236 (3) Å	Cell parameters from 5176 reflections
*b* = 9.6807 (3) Å	θ = 2.2–31.3°
*c* = 14.3345 (5) Å	µ = 0.18 mm^−^^1^
α = 87.939 (2)°	*T* = 293 K
β = 89.680 (2)°	Block, yellow
γ = 75.287 (2)°	0.47 × 0.35 × 0.20 mm
*V* = 1129.86 (7) Å^3^	

### Data collection

**Table:**

Bruker SMART APEX CCD diffractometer	3969 independent reflections
Radiation source: fine-focus sealed tube	3438 reflections with *I* > 2σ(*I*)
graphite	*R*_int_ = 0.024
ω and φ scans	θ_max_ = 25.0°, θ_min_ = 1.4°
Absorption correction: multi-scan (*SADABS*; Sheldrick, 1996)	*h* = −10→10
*T*_min_ = 0.920, *T*_max_ = 0.965	*k* = −11→11
20701 measured reflections	*l* = −17→17

### Refinement

**Table:**

Refinement on *F*^2^	Primary atom site location: structure-invariant direct methods
Least-squares matrix: full	Secondary atom site location: difference Fourier map
*R*[*F*^2^ > 2σ(*F*^2^)] = 0.044	Hydrogen site location: inferred from neighbouring sites
*wR*(*F*^2^) = 0.137	H-atom parameters constrained
*S* = 1.06	*w* = 1/[σ^2^(*F*_o_^2^) + (0.0721*P*)^2^ + 0.4966*P*] where *P* = (*F*_o_^2^ + 2*F*_c_^2^)/3
3969 reflections	(Δ/σ)_max_ = 0.002
301 parameters	Δρ_max_ = 0.36 e Å^−^^3^
0 restraints	Δρ_min_ = −0.31 e Å^−^^3^

### Special details

**Table:**

Geometry. All s.u.'s (except the s.u. in the dihedral angle between two l.s. planes) are estimated using the full covariance matrix. The cell s.u.'s are taken into account individually in the estimation of s.u.'s in distances, angles and torsion angles; correlations between s.u.'s in cell parameters are only used when they are defined by crystal symmetry. An approximate (isotropic) treatment of cell s.u.'s is used for estimating s.u.'s involving l.s. planes.
Refinement. Refinement of *F*^2^ against ALL reflections. The weighted *R*-factor wR and goodness of fit *S* are based on *F*^2^, conventional *R*-factors *R* are based on *F*, with *F* set to zero for negative *F*^2^. The threshold expression of *F*^2^ > 2σ(*F*^2^) is used only for calculating *R*-factors(gt) etc. and is not relevant to the choice of reflections for refinement. *R*-factors based on *F*^2^ are statistically about twice as large as those based on *F*, and *R*- factors based on ALL data will be even larger.

### Fractional atomic coordinates and isotropic or equivalent isotropic displacement parameters (Å^2^)

**Table:**

	*x*	*y*	*z*	*U*_iso_*/*U*_eq_	
S1	0.19582 (7)	0.35035 (6)	0.10853 (4)	0.0550 (2)	
O1	0.8697 (2)	0.4977 (2)	0.28339 (16)	0.0886 (6)	
O2	0.87965 (18)	0.27940 (18)	0.33559 (11)	0.0604 (4)	
O3	0.44199 (18)	0.03161 (15)	0.25695 (11)	0.0563 (4)	
O4	−0.00662 (19)	−0.14186 (16)	0.17321 (11)	0.0590 (4)	
O5	−0.1285 (3)	−0.0711 (3)	0.30616 (14)	0.1097 (9)	
N1	0.4187 (2)	0.4897 (2)	0.14656 (13)	0.0568 (5)	
N2	0.45080 (18)	0.25926 (16)	0.21508 (10)	0.0387 (4)	
C1	0.3732 (2)	0.3754 (2)	0.16117 (13)	0.0447 (5)	
C2	0.5865 (2)	0.2654 (2)	0.27852 (12)	0.0383 (4)	
H2	0.6743	0.1775	0.2736	0.046*	
C3	0.6520 (2)	0.3922 (2)	0.24663 (13)	0.0433 (4)	
C4	0.5692 (3)	0.4949 (2)	0.18730 (15)	0.0524 (5)	
C5	0.5251 (2)	0.2766 (2)	0.37843 (12)	0.0377 (4)	
C6	0.4075 (3)	0.3956 (2)	0.40504 (15)	0.0496 (5)	
H6	0.3675	0.4700	0.3618	0.060*	
C7	0.3486 (3)	0.4053 (3)	0.49501 (17)	0.0640 (6)	
H7	0.2689	0.4858	0.5121	0.077*	
C8	0.4071 (3)	0.2968 (3)	0.55903 (16)	0.0664 (7)	
H8	0.3666	0.3029	0.6196	0.080*	
C9	0.5252 (4)	0.1794 (3)	0.53393 (16)	0.0690 (7)	
H9	0.5663	0.1062	0.5779	0.083*	
C10	0.5843 (3)	0.1683 (2)	0.44344 (15)	0.0552 (5)	
H10	0.6640	0.0875	0.4267	0.066*	
C11	0.8095 (3)	0.3985 (2)	0.28807 (14)	0.0492 (5)	
C12	1.0308 (3)	0.2780 (3)	0.38364 (18)	0.0651 (6)	
H12A	1.1119	0.2944	0.3392	0.078*	
H12B	1.0113	0.3533	0.4284	0.078*	
C13	1.0905 (4)	0.1404 (4)	0.4313 (3)	0.1057 (12)	
H13A	1.0139	0.1285	0.4789	0.158*	
H13B	1.1952	0.1352	0.4593	0.158*	
H13C	1.1019	0.0661	0.3872	0.158*	
C14	0.6204 (4)	0.6257 (3)	0.1538 (2)	0.0819 (9)	
H14A	0.7278	0.6218	0.1777	0.123*	
H14B	0.5436	0.7094	0.1755	0.123*	
H14C	0.6227	0.6296	0.0868	0.123*	
C15	0.3839 (2)	0.1429 (2)	0.21508 (13)	0.0409 (4)	
C16	0.2306 (2)	0.1780 (2)	0.15845 (12)	0.0417 (4)	
C17	0.1391 (2)	0.0840 (2)	0.15492 (13)	0.0445 (4)	
H17	0.1848	−0.0036	0.1852	0.053*	
C18	−0.0194 (2)	0.0952 (2)	0.11188 (13)	0.0437 (4)	
C19	−0.0947 (2)	−0.0168 (2)	0.12602 (14)	0.0463 (5)	
C20	−0.2463 (3)	−0.0123 (3)	0.08936 (16)	0.0563 (5)	
H20	−0.2943	−0.0877	0.1009	0.068*	
C21	−0.3265 (3)	0.1051 (3)	0.03518 (16)	0.0563 (6)	
H21	−0.4290	0.1089	0.0099	0.068*	
C22	−0.2554 (3)	0.2161 (3)	0.01856 (15)	0.0555 (5)	
H22	−0.3092	0.2948	−0.0184	0.067*	
C23	−0.1045 (3)	0.2112 (2)	0.05660 (15)	0.0516 (5)	
H23	−0.0580	0.2876	0.0450	0.062*	
C24	−0.0289 (3)	−0.1560 (3)	0.26542 (18)	0.0612 (6)	
C25	0.0891 (4)	−0.2840 (3)	0.3062 (2)	0.0810 (8)	
H25A	0.1858	−0.2582	0.3265	0.122*	
H25B	0.1185	−0.3553	0.2599	0.122*	
H25C	0.0396	−0.3214	0.3585	0.122*	

### Atomic displacement parameters (Å^2^)

**Table:**

	*U*^11^	*U*^22^	*U*^33^	*U*^12^	*U*^13^	*U*^23^
S1	0.0523 (3)	0.0583 (4)	0.0560 (3)	−0.0191 (3)	−0.0217 (2)	0.0164 (3)
O1	0.0699 (12)	0.0833 (13)	0.1248 (17)	−0.0447 (11)	−0.0285 (11)	0.0212 (12)
O2	0.0430 (8)	0.0730 (11)	0.0683 (10)	−0.0222 (7)	−0.0161 (7)	0.0111 (8)
O3	0.0549 (9)	0.0473 (8)	0.0666 (10)	−0.0140 (7)	−0.0186 (7)	0.0120 (7)
O4	0.0563 (9)	0.0499 (9)	0.0684 (10)	−0.0095 (7)	−0.0008 (7)	−0.0001 (7)
O5	0.1234 (19)	0.1067 (17)	0.0643 (12)	0.0321 (15)	0.0107 (12)	0.0131 (11)
N1	0.0579 (11)	0.0560 (11)	0.0592 (11)	−0.0222 (9)	−0.0177 (9)	0.0186 (9)
N2	0.0374 (8)	0.0434 (9)	0.0353 (8)	−0.0103 (7)	−0.0055 (6)	0.0019 (6)
C1	0.0444 (11)	0.0503 (11)	0.0394 (10)	−0.0129 (9)	−0.0074 (8)	0.0067 (8)
C2	0.0333 (9)	0.0426 (10)	0.0384 (9)	−0.0083 (7)	−0.0053 (7)	0.0013 (8)
C3	0.0408 (10)	0.0516 (11)	0.0400 (10)	−0.0163 (9)	0.0025 (8)	−0.0018 (8)
C4	0.0555 (12)	0.0577 (13)	0.0483 (11)	−0.0237 (10)	−0.0045 (9)	0.0095 (9)
C5	0.0342 (9)	0.0437 (10)	0.0373 (9)	−0.0139 (8)	−0.0059 (7)	0.0011 (8)
C6	0.0470 (11)	0.0524 (12)	0.0470 (11)	−0.0083 (9)	−0.0027 (9)	−0.0003 (9)
C7	0.0581 (14)	0.0772 (16)	0.0566 (14)	−0.0151 (12)	0.0098 (11)	−0.0174 (12)
C8	0.0754 (16)	0.0904 (19)	0.0425 (12)	−0.0371 (15)	0.0078 (11)	−0.0091 (12)
C9	0.0877 (18)	0.0779 (17)	0.0437 (12)	−0.0279 (15)	−0.0091 (12)	0.0177 (11)
C10	0.0588 (13)	0.0547 (13)	0.0485 (12)	−0.0088 (10)	−0.0059 (10)	0.0078 (10)
C11	0.0420 (11)	0.0611 (13)	0.0471 (11)	−0.0179 (10)	0.0036 (9)	−0.0022 (10)
C12	0.0417 (12)	0.0902 (18)	0.0647 (15)	−0.0188 (12)	−0.0125 (10)	−0.0020 (13)
C13	0.088 (2)	0.102 (2)	0.128 (3)	−0.0310 (19)	−0.055 (2)	0.030 (2)
C14	0.092 (2)	0.0854 (19)	0.0819 (18)	−0.0525 (17)	−0.0265 (15)	0.0380 (15)
C15	0.0426 (10)	0.0446 (11)	0.0357 (9)	−0.0116 (8)	−0.0018 (8)	−0.0004 (8)
C16	0.0420 (10)	0.0487 (11)	0.0336 (9)	−0.0100 (8)	−0.0038 (7)	−0.0010 (8)
C17	0.0452 (11)	0.0487 (11)	0.0399 (10)	−0.0125 (9)	−0.0057 (8)	0.0006 (8)
C18	0.0439 (10)	0.0515 (11)	0.0365 (9)	−0.0129 (9)	−0.0018 (8)	−0.0051 (8)
C19	0.0461 (11)	0.0487 (11)	0.0443 (11)	−0.0118 (9)	−0.0001 (8)	−0.0040 (9)
C20	0.0498 (12)	0.0614 (14)	0.0629 (13)	−0.0231 (10)	−0.0017 (10)	−0.0055 (11)
C21	0.0424 (11)	0.0719 (15)	0.0565 (13)	−0.0172 (10)	−0.0071 (9)	−0.0070 (11)
C22	0.0530 (12)	0.0635 (14)	0.0487 (12)	−0.0126 (11)	−0.0120 (9)	0.0030 (10)
C23	0.0533 (12)	0.0564 (12)	0.0483 (11)	−0.0205 (10)	−0.0099 (9)	0.0038 (9)
C24	0.0591 (14)	0.0552 (14)	0.0684 (15)	−0.0133 (11)	−0.0091 (12)	0.0029 (11)
C25	0.0785 (18)	0.0565 (15)	0.105 (2)	−0.0147 (13)	−0.0237 (16)	0.0202 (14)

### Geometric parameters (Å, °)

**Table:**

S1—C16	1.747 (2)	C9—H9	0.9300
S1—C1	1.752 (2)	C10—H10	0.9300
O1—C11	1.194 (3)	C12—C13	1.447 (4)
O2—C11	1.321 (3)	C12—H12A	0.9700
O2—C12	1.448 (3)	C12—H12B	0.9700
O3—C15	1.204 (2)	C13—H13A	0.9600
O4—C24	1.341 (3)	C13—H13B	0.9600
O4—C19	1.402 (2)	C13—H13C	0.9600
O5—C24	1.183 (3)	C14—H14A	0.9600
N1—C1	1.270 (3)	C14—H14B	0.9600
N1—C4	1.412 (3)	C14—H14C	0.9600
N2—C1	1.363 (2)	C15—C16	1.487 (3)
N2—C15	1.382 (2)	C16—C17	1.336 (3)
N2—C2	1.479 (2)	C17—C18	1.451 (3)
C2—C5	1.518 (2)	C17—H17	0.9300
C2—C3	1.523 (3)	C18—C23	1.391 (3)
C2—H2	0.9800	C18—C19	1.397 (3)
C3—C4	1.339 (3)	C19—C20	1.373 (3)
C3—C11	1.473 (3)	C20—C21	1.380 (3)
C4—C14	1.501 (3)	C20—H20	0.9300
C5—C10	1.373 (3)	C21—C22	1.370 (3)
C5—C6	1.379 (3)	C21—H21	0.9300
C6—C7	1.377 (3)	C22—C23	1.375 (3)
C6—H6	0.9300	C22—H22	0.9300
C7—C8	1.364 (4)	C23—H23	0.9300
C7—H7	0.9300	C24—C25	1.481 (3)
C8—C9	1.365 (4)	C25—H25A	0.9600
C8—H8	0.9300	C25—H25B	0.9600
C9—C10	1.385 (3)	C25—H25C	0.9600
			
C16—S1—C1	91.39 (9)	C12—C13—H13A	109.5
C11—O2—C12	116.05 (18)	C12—C13—H13B	109.5
C24—O4—C19	118.48 (17)	H13A—C13—H13B	109.5
C1—N1—C4	116.62 (18)	C12—C13—H13C	109.5
C1—N2—C15	116.40 (16)	H13A—C13—H13C	109.5
C1—N2—C2	121.05 (16)	H13B—C13—H13C	109.5
C15—N2—C2	122.13 (15)	C4—C14—H14A	109.5
N1—C1—N2	126.93 (18)	C4—C14—H14B	109.5
N1—C1—S1	121.26 (15)	H14A—C14—H14B	109.5
N2—C1—S1	111.80 (14)	C4—C14—H14C	109.5
N2—C2—C5	109.63 (14)	H14A—C14—H14C	109.5
N2—C2—C3	107.89 (15)	H14B—C14—H14C	109.5
C5—C2—C3	112.53 (15)	O3—C15—N2	123.80 (17)
N2—C2—H2	108.9	O3—C15—C16	126.21 (18)
C5—C2—H2	108.9	N2—C15—C16	109.98 (16)
C3—C2—H2	108.9	C17—C16—C15	119.85 (18)
C4—C3—C11	121.68 (19)	C17—C16—S1	129.76 (16)
C4—C3—C2	122.34 (18)	C15—C16—S1	110.33 (14)
C11—C3—C2	115.93 (17)	C16—C17—C18	130.97 (19)
C3—C4—N1	122.26 (19)	C16—C17—H17	114.5
C3—C4—C14	126.5 (2)	C18—C17—H17	114.5
N1—C4—C14	111.25 (19)	C23—C18—C19	116.47 (18)
C10—C5—C6	118.94 (18)	C23—C18—C17	124.65 (19)
C10—C5—C2	120.72 (17)	C19—C18—C17	118.87 (18)
C6—C5—C2	120.33 (17)	C20—C19—C18	122.2 (2)
C7—C6—C5	120.7 (2)	C20—C19—O4	119.12 (19)
C7—C6—H6	119.6	C18—C19—O4	118.50 (18)
C5—C6—H6	119.6	C19—C20—C21	119.4 (2)
C8—C7—C6	120.0 (2)	C19—C20—H20	120.3
C8—C7—H7	120.0	C21—C20—H20	120.3
C6—C7—H7	120.0	C22—C21—C20	120.1 (2)
C7—C8—C9	119.8 (2)	C22—C21—H21	119.9
C7—C8—H8	120.1	C20—C21—H21	119.9
C9—C8—H8	120.1	C21—C22—C23	120.0 (2)
C8—C9—C10	120.5 (2)	C21—C22—H22	120.0
C8—C9—H9	119.8	C23—C22—H22	120.0
C10—C9—H9	119.8	C22—C23—C18	121.9 (2)
C5—C10—C9	120.0 (2)	C22—C23—H23	119.1
C5—C10—H10	120.0	C18—C23—H23	119.1
C9—C10—H10	120.0	O5—C24—O4	121.8 (2)
O1—C11—O2	121.8 (2)	O5—C24—C25	126.7 (3)
O1—C11—C3	126.4 (2)	O4—C24—C25	111.4 (2)
O2—C11—C3	111.81 (18)	C24—C25—H25A	109.5
C13—C12—O2	108.6 (2)	C24—C25—H25B	109.5
C13—C12—H12A	110.0	H25A—C25—H25B	109.5
O2—C12—H12A	110.0	C24—C25—H25C	109.5
C13—C12—H12B	110.0	H25A—C25—H25C	109.5
O2—C12—H12B	110.0	H25B—C25—H25C	109.5
H12A—C12—H12B	108.4		
			
C4—N1—C1—N2	2.8 (3)	C12—O2—C11—C3	−176.56 (17)
C4—N1—C1—S1	−175.81 (16)	C4—C3—C11—O1	9.3 (4)
C15—N2—C1—N1	−175.5 (2)	C2—C3—C11—O1	−168.3 (2)
C2—N2—C1—N1	11.7 (3)	C4—C3—C11—O2	−172.59 (19)
C15—N2—C1—S1	3.2 (2)	C2—C3—C11—O2	9.8 (2)
C2—N2—C1—S1	−169.56 (13)	C11—O2—C12—C13	−179.7 (2)
C16—S1—C1—N1	177.52 (19)	C1—N2—C15—O3	177.60 (18)
C16—S1—C1—N2	−1.30 (15)	C2—N2—C15—O3	−9.7 (3)
C1—N2—C2—C5	103.91 (19)	C1—N2—C15—C16	−3.7 (2)
C15—N2—C2—C5	−68.5 (2)	C2—N2—C15—C16	168.97 (15)
C1—N2—C2—C3	−18.9 (2)	O3—C15—C16—C17	3.7 (3)
C15—N2—C2—C3	168.69 (16)	N2—C15—C16—C17	−174.93 (17)
N2—C2—C3—C4	15.1 (3)	O3—C15—C16—S1	−178.82 (17)
C5—C2—C3—C4	−106.0 (2)	N2—C15—C16—S1	2.55 (19)
N2—C2—C3—C11	−167.40 (15)	C1—S1—C16—C17	176.4 (2)
C5—C2—C3—C11	71.5 (2)	C1—S1—C16—C15	−0.73 (14)
C11—C3—C4—N1	179.53 (19)	C15—C16—C17—C18	174.58 (18)
C2—C3—C4—N1	−3.1 (3)	S1—C16—C17—C18	−2.3 (3)
C11—C3—C4—C14	0.7 (4)	C16—C17—C18—C23	5.5 (3)
C2—C3—C4—C14	178.1 (2)	C16—C17—C18—C19	−174.4 (2)
C1—N1—C4—C3	−7.1 (3)	C23—C18—C19—C20	−1.4 (3)
C1—N1—C4—C14	171.9 (2)	C17—C18—C19—C20	178.47 (19)
N2—C2—C5—C10	114.6 (2)	C23—C18—C19—O4	173.64 (17)
C3—C2—C5—C10	−125.3 (2)	C17—C18—C19—O4	−6.5 (3)
N2—C2—C5—C6	−64.7 (2)	C24—O4—C19—C20	−89.8 (2)
C3—C2—C5—C6	55.3 (2)	C24—O4—C19—C18	94.9 (2)
C10—C5—C6—C7	−0.7 (3)	C18—C19—C20—C21	1.3 (3)
C2—C5—C6—C7	178.63 (19)	O4—C19—C20—C21	−173.78 (19)
C5—C6—C7—C8	0.3 (4)	C19—C20—C21—C22	−0.2 (3)
C6—C7—C8—C9	0.6 (4)	C20—C21—C22—C23	−0.6 (3)
C7—C8—C9—C10	−1.1 (4)	C21—C22—C23—C18	0.4 (3)
C6—C5—C10—C9	0.3 (3)	C19—C18—C23—C22	0.6 (3)
C2—C5—C10—C9	−179.1 (2)	C17—C18—C23—C22	−179.28 (19)
C8—C9—C10—C5	0.6 (4)	C19—O4—C24—O5	5.5 (4)
C12—O2—C11—O1	1.7 (3)	C19—O4—C24—C25	−172.2 (2)

### Hydrogen-bond geometry (Å, °)

**Table:**

Cg1 is the centroid of the S1/C1/N2/C15/C16 ring.

**Table:**

*D*—H···*A*	*D*—H	H···*A*	*D*···*A*	*D*—H···*A*
C2—H2···O5^i^	0.98	2.58	3.532 (3)	163
C12—H12a···Cg1^i^	0.97	2.98	3.902 (3)	160

Symmetry codes: (i) *x*+1, *y*, *z*.
